# Dopamine Replacement Therapy, Learning and Reward Prediction in Parkinson’s Disease: Implications for Rehabilitation

**DOI:** 10.3389/fnbeh.2016.00121

**Published:** 2016-06-14

**Authors:** Davide Ferrazzoli, Adrian Carter, Fatma S. Ustun, Grazia Palamara, Paola Ortelli, Roberto Maestri, Murat Yücel, Giuseppe Frazzitta

**Affiliations:** ^1^Department of Parkinson’s disease, Movement Disorders and Brain Injury Rehabilitation, “Moriggia-Pelascini” HospitalGravedona ed Uniti (Como), Italy; ^2^UQ Centre for Clinical Research, The University of QueenslandBrisbane, QLD, Australia; ^3^School of Psychological Sciences and Monash Institute of Cognitive and Clinical Neurosciences, Monash UniversityMelbourne, VIC, Australia; ^4^Neuroscience Graduate Program and National Magnetic Resonance Research Center (UMRAM), Bilkent UniversityAnkara, Turkey; ^5^Department of Biomedical Engineering, Scientific Institute of Montescano, S. Maugeri Foundation, Istituto di Ricovero e Cura a Carattere Scientifico (IRCCS)Montescano (Pavia), Italy

**Keywords:** dopamine replacement therapy, rehabilitation, learning, reward prediction, DRT side effects

## Abstract

The principal feature of Parkinson’s disease (PD) is the impaired ability to acquire and express habitual-automatic actions due to the loss of dopamine in the dorsolateral striatum, the region of the basal ganglia associated with the control of habitual behavior. Dopamine replacement therapy (DRT) compensates for the lack of dopamine, representing the standard treatment for different motor symptoms of PD (such as rigidity, bradykinesia and resting tremor). On the other hand, rehabilitation treatments, exploiting the use of cognitive strategies, feedbacks and external cues, permit to “learn to bypass” the defective basal ganglia (using the dorsolateral area of the prefrontal cortex) allowing the patients to perform correct movements under executive-volitional control. Therefore, DRT and rehabilitation seem to be two complementary and synergistic approaches. Learning and reward are central in rehabilitation: both of these mechanisms are the basis for the success of any rehabilitative treatment. Anyway, it is known that “learning resources” and reward could be negatively influenced from dopaminergic drugs. Furthermore, DRT causes different well-known complications: among these, dyskinesias, motor fluctuations, and dopamine dysregulation syndrome (DDS) are intimately linked with the alteration in the learning and reward mechanisms and could impact seriously on the rehabilitative outcomes. These considerations highlight the need for careful titration of DRT to produce the desired improvement in motor symptoms while minimizing the associated detrimental effects. This is important in order to maximize the motor re-learning based on repetition, reward and practice during rehabilitation. In this scenario, we review the knowledge concerning the interactions between DRT, learning and reward, examine the most impactful DRT side effects and provide suggestions for optimizing rehabilitation in PD.

## Introduction

Parkinson’s disease (PD) is a progressive neurodegenerative disorder clinically dominated by bradykinesia, rigidity and resting tremor. The neuropathological hallmark of PD is the dopaminergic neuronal loss in the pars compacta of the substantia nigra (Less et al., 2009). The loss of the physiological dopaminergic modulation alters the cortico-striatal plasticity and transforms the basal ganglia into a disruptive filter (Beeler et al., 2013) that impairs the ability to acquire and express habitual-automatic movements (Redgrave et al., 2010). Dopamine replacement therapy (DRT) is the standard treatment for the motor symptoms of PD: the dopamine precursor levodopa (L-DOPA), dopamine agonists (DAs), monoamine oxidase B inhibitors and catechol-O-methyltrasferase inhibitors, are commonly used. Long-term DRT is able to restore the physiological synaptic plasticity in the dopamine-denervated striatum (Calabresi et al., 2015) but might also cause, by itself, aberrant structural plasticity in the striatal medium spiny neurons causing further functional short and long term alterations of neural transmission (Nishijima et al., 2014).

In recent years, rehabilitation has been proposed as effective and complementary treatment for the management of PD (Tomlinson et al., 2013; Bloem et al., 2015). Indeed, exercise may influence neuroplasticity through activity-dependent processes in the basal ganglia acting on dopaminergic and glutamatergic neurotransmission (Petzinger et al., 2010). The great value of rehabilitation is the possibility to treat many disabling PD disturbances (such as balance dysfunctions, postural instability and freezing of gait) that do not respond to DRT as they result from the involvement of systems outside the dopaminergic structures (e.g., cholinergic, serotoninergic, and noradrenergic; Calabresi et al., 2013). Rehabilitation exploiting the use of cognitive strategies, feedbacks and external cues, permits to bypass the defective basal ganglia using the dorsolateral area of the prefrontal cortex and allowing the execution of correct movements under executive-volitional control (Morris, 2006; Morris et al., 2009). This is possible because motor learning is feasible in PD subjects, as argued in previous studies (Nieuwboer et al., 2009). Nevertheless, the long-term DRT treatment, inducing aberrant structural effects on cortico-striatal plasticity, could negatively influence the learning process with the related motivational components subtended by reward mechanisms (Swainson et al., 2000; Cools et al., 2006; Shohamy et al., 2006; Jahanshahi et al., 2010; Claassen et al., 2011; Voon et al., 2011a; Steinberg et al., 2013; Fuhrer et al., 2014).

Furthermore, DRT causes different well-known complications: among these, dyskinesias (Obeso et al., 2005), motor fluctuations (Kikuchi, 2007), and dopamine dysregulation syndrome (DDS; Lawrence et al., 2003) could impact seriously on the rehabilitative outcomes. These side effects are intimately linked with the alteration in the learning and reward mechanisms: striatum-dependent learning functions and striatal reward-related motivational processes are affected in patients with dyskinesias/motor fluctuations and DDS respectively (Feigin et al., 2003; Cenci and Konradi, 2010).

Learning and reward are central in the motor-cognitive rehabilitation and the integrity of both of these mechanisms, as well as the control of the DRT side effects, are the basis for the success of any rehabilitative approach in PD. Therefore, given these premises, optimizing drug titration in PD may be critical for disease management and for good rehabilitative outcome.

In this article, after discussing the key points for effectiveness of PD rehabilitation, we review the knowledge concerning the interactions between DRT, learning and reward, examine the most impactful DRT side effects and provide suggestions for optimizing rehabilitation in PD.

## Rehabilitation in PD: Key Points for Effectiveness

Exercise is beneficial in PD since is able to promote the so-called “activity-dependent neuroplasticity” (Petzinger et al., 2010; Frazzitta et al., 2013a). It is defined as modifications within the central nervous system (CNS) in response to physical activity that promote a skill acquisition process (Adkins et al., 2006). Mechanisms by which exercise lead to these beneficial effects may be through mitigating the pathological hyperexcitability in basal ganglia cortical circuits and inducing compensatory changes in dopamine handling and neurotransmission (Petzinger et al., 2007). Interestingly, this exercise effect in dopamine release is most pronounced properly in the dorsolateral striatum, associated with the control of habitual behavior (Petzinger et al., 2007, 2013). Intensity, specificity, difficulty and complexity of practice appear to be important parameters for driving this phenomenon (Petzinger et al., 2010). Petzinger et al. (2007) in a mice model of PD found that high intensity exercise determines improvements in running velocity, endurance and on a motor task designed to assess balance. A significant clinical improvement after intensive and goal-based rehabilitation treatment was also found in humans (Frazzitta et al., 2015), confirming that specific motor training could exploit the physiological mechanisms of neural plasticity (Petzinger et al., 2013; Frazzitta et al., 2014; Fontanesi et al., 2015).

The bases for effective rehabilitation involve several important recommendations that are consistent with model. These authors Fitts and Posner’s (1967) proposed that the capability to learn a motor skill involves three stages: (1) a “cognitive stage” that involves executive functions for identification and development of different parts of the movement with formation of a mental-motor schema; (2) an “associative stage” built on reward-based decision-making, in which specific environmental feedbacks are required to achieve the goal; and (3) an “autonomous stage” in which automaticity is achieved.

Different brain circuits, including prefrontal cortex and basal ganglia, are involved in reward-based decision-making and motor learning, and dopamine plays a modulatory role in these functions (Schultz, 2002). Motor learning in PD is negatively affected throughout the automatization phase (Doyon et al., 2009; Nieuwboer et al., 2009) Given that, the main purpose of rehabilitation in PD should be the re-acquisition of the lost automatic movements through executive-volitional control that is the ability to initiate habits using goal-directed triggers.

On these bases, Morris et al. (2010) showed that external cues, such as lines on the floor, visualizing the walk with long steps, imagining the movement pattern before the action is performed, breaking down long or complex motor sequences into parts, enabled people with PD to walk with longer steps and at a more normal stepping rate, reducing move and balance difficulty (Morris et al., 1994, 1996, 2009; Morris, 2006). Furthermore, the use of treadmill, acting as an external cue, enhances gait rhythmicity and reduces gait variability improving walking in Parkinsonian patients (Frenkel-Toledo et al., 2005; Frazzitta et al., 2009, 2013b). These strategies exploits executive functions and represent the basis to re-learn motor skills in PD (Nieuwboer et al., 2009).

Further, in order to maximize learning, is crucial the ability to associate stimuli with actions that lead to rewarding outcomes (van Wouwe et al., 2012). Therefore, in the following sections, we will explore the impact of DRT on learning and reward.

## The Impact of DRT on Learning and Reward

**Figure 1:**
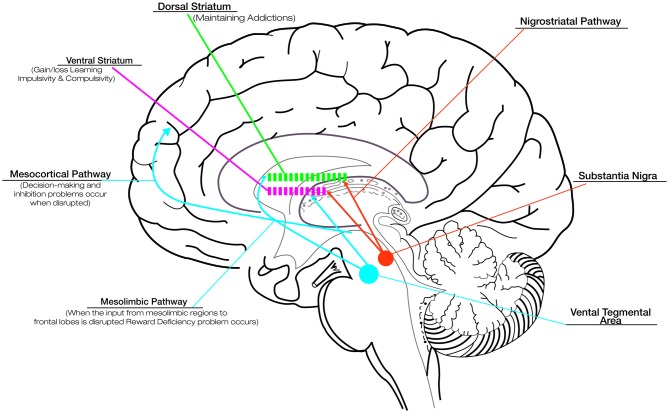
**Dopaminergic pathways and the role of dopamine in reward, compulsive behavior and addiction.** Although dopamine replacement therapy (DRT) successfully improves motor deficits in Parkinson’s disease (PD), its effects on cognitive and motivational processes are more equivocal. This is related to the effect that DRT exercises on the different dopaminergic pathways: the nigrostriatal pathway, responsible for voluntary movements, connects the substantia nigra with the dorsal striatum; the mesocorticolimbic pathways, associated with reward and motivation-related processes, connect the ventral tegmental area to the ventral striatum and the cerebral cortex (in particular the frontal lobes). In the early stages of PD the ventral striatal dopamine projections are relatively preserved, and the dopaminergic drugs lead to an “overflow” of dopamine in the ventral striatum and mesocorticolimbic system. This overflow is responsible for the problems in reward (due to disruption of the dopaminergic input to mesolimbic pathway), motivational-related processing and maladaptive decision-making (due to disruption of the dopaminergic input to mesocortical pathway), impaired ability to learn from negative consequences with compulsive behaviors and impulsivity (due to alteration of dopaminergic input to ventral striatum). Drug addiction, related to the disruption of the dopaminergic input to dorsal striatum represents a pathological shift form voluntary drug use to more habitual and compulsive drug intake. The alteration in the learning and reward mechanisms could impact seriously on the rehabilitative outcomes. Thus, it is necessary to titrate carefully DRT to produce the desired improvement in motor symptoms while minimizing the associated detrimental effects.

Although the basal ganglia are traditionally known to contribute to motor function, more recently they have been shown to be engaged in several types of learning, including habit formation, procedural skill learning, and reward-based decision-learning (van Wouwe et al., 2012). Therefore, involving the disruption of basal ganglia, PD provides an informative naturalistic model for understanding the role of dopamine in reward and learning. Although DRT successfully improves motor deficits in PD, its effects on cognitive and motivational processes are more equivocal. The fact that DRT acts differentially on such circuits has been explained in terms of an “overdose theory” (Cools, 2006; Torta et al., 2009). There are two main dopamine pathways in the brain: (1) the nigrostriatal pathway, responsible for voluntary movements, is the target area for treating motor symptoms in PD and is associated with activation in dorsolateral striatum; and (2) the mesocorticolimbic pathway, strongly associated with reward and motivation-related processes, involves the activation of the ventral portions of the striatum (see Figure 1; Wise, 2009). In the early stages of PD the ventral striatal dopamine projections are relatively preserved, and the dopaminergic drugs lead to an “overflow” of dopamine in the ventral striatum and mesocorticolimbic system. This fact causes problems in reward and in the motivational-related processing (Claassen et al., 2011; Voon et al., 2011a), leading to maladaptive decision-making and compulsive behaviors (Swainson et al., 2000). While DRT ameliorates the task associated with the dorsal fronto-striatal circuitry, such as cognitive flexibility (Cools, 2006), the excessive levels of dopamine in mesocorticolimbic system alter striatal reward prediction error (RPE) activity, i.e., the difference between expected and actual reward. The RPE signal is needed to optimize behavior and learning. In physiological conditions, a negative RPE is conveyed by pauses in dopamine neuron firing (Bayer et al., 2007). Instead, in medicated PD patients, persistent postsynaptic dopamine stimulation may reduce the ability of these pauses to influence learning, impairing the ability to learn from negative consequences and resulting in increased engagement in reward-seeking behaviors (Frank et al., 2004; Cools et al., 2006).

In this scenario, it has also been found that DRT has a detrimental effect on habit learning (Fuhrer et al., 2014), on error-correcting and feedback-based learning processes (Shohamy et al., 2006) and impairs reversal shifting in those conditions where reversals are signaled by unexpected punishment (Cools et al., 2006). The deterioration of these forms of learning supports the proposal that tonic dopaminergic increase masks phasic changes in dopamine release essential for learning in medicated PD patients (Jahanshahi et al., 2010).

## The Impact of DRT Side Effects

The most impactful DRT side effects consist in dyskinesias, motor fluctuations and DDS. Common neurophysiopathological mechanisms involving the cortico-striatal plasticity link these motor and behavioral side effects (Voon et al., 2009, 2011a,b).

*Dyskinesias* refer to a category of movement disorders that are characterized by involuntary muscle movements*.* They occur in more than half of PD patients after 5–10 years of L-DOPA treatment, with the percentage of affected patients increasing over time (Obeso et al., 2005). They commonly present as chorea or choreoathetosis, though myoclonus, akathasia, ballism and other forms of abnormal movements. They lead to exhaustion, fatigue, risk of injury and weight loss causing pronounced discomfort and physical limitation. If dyskinesias becomes too severe impair the patient’s quality of life and a reduction in L-DOPA might be necessary.

*Motor fluctuations*, such as wearing-off and *on*–*off* effect, are approximately experienced by 40% after 4–6 years of treatment with L-DOPA, similar to the frequency of dyskinesias (Kikuchi, 2007). Wearing off is the most common motor fluctuation seen in patients with PD. With this pattern, patients develop a predictable worsening of their parkinsonism at the end of the dose because of the short duration benefit after a given dose of L-DOPA. With disease progression the dosing response varies and patients may report a “delayed-on” or “no-on” (Kikuchi, 2007). On-off fluctuations are characterized by sudden and unpredictable shifts between on and off state.

*DDS* represents a pattern of addictive drug use: it occurs in 3–4% of PD patients treated with DRT although this phenomenon is probably under-diagnosed (Merims and Giladi, 2008). DDS is predominately reported in patients receiving L-DOPA, but the development of this “addiction-like” behavior has been found also in patients using DAs and apomorphine (Giovannoni et al., 2000; Lawrence et al., 2003; Witjas et al., 2005; Merims and Giladi, 2008). Parkinsonian patients with DDS rapidly develop a pattern of compulsive DRT-seeking, leading to the intake of high daily L-DOPA doses. Patients eagerly wait for ignition of the “on” period after an oral dose of L-DOPA (Merims and Giladi, 2008). In the “off” period, they are often agitated, depressed and anxious resembling withdrawal phenomena exhibited by drug addicts (Hughes et al., 1994). In extreme cases, patients will report only feeling “on” and “mobile” when notably dyskinetic and deliberately exaggerate the description of their clinical status to get more medication from the doctor (Lawrence et al., 2003).

## Optimising Rehabilitation in PD

As argued before, the rehabilitative interventions in PD should be based on repetition and practice: several intensive and specific approaches drive the re-acquisition of the lost automatic movements exploiting executive functions and the ability to initiate habits using goal-directed triggers (a summary of the aims of rehabilitation on PD symptoms may be found in Table 1). Frazzitta et al. (2012b, 2015) demonstrated that a multidisciplinary and intensive rehabilitative treatment, designed according to these concepts, acts positively on early PD, possibly influencing the natural progression of motor impairment and reducing the need for increasing DRT. The patients enrolled in these studies underwent very intensive and goal-based treatment that included physical exercises and the use of a number of devices, such as a stabilometric platform and a treadmill with auditory and visual cues (Frazzitta et al., 2009). Similarly, Ellis et al. (2008) studied in inpatients with PD the efficacy of an intensive program: after treatment, all patients showed significant improvements in motor and functional evaluated outcomes. Corcos et al. (2013) reported that progressive resistance exercise improved motor performances in PD patients with an effect lasting up to 2 years. Shulman et al. (2013) suggested the importance of a combination of treadmill training and resistance exercise to obtain a greater benefit in patients with PD. According to all these findings, Fisher et al. (2008) previously showed that high intensity exercise could normalize corticomotor excitability (assessed with cortical silent period durations in response to single-pulse transcranial magnetic stimulation) in early PD, suggesting that high-intensity exercise may induce activity-dependent neuroplasticity.

**Table 1:** **The aims of rehabilitation on Parkinson’s disease (PD) symptoms**.

Aim	Implications for Rehabilitation
Reacquisition of automatic movements	Teaching to utilize PFC instead of basal ganglia through volitional control to regulate movement
Improve impaired ability to acquire and express habitual-automatic actions	Repetition and practice of actions supported by DRT management
Motor learning	Motor learning-based rehabilitation in addition to DRT
Avoid compulsive DRT use	Rehabilitation in off period using correct levodopa dose

*Abbreviations: DRT, dopamine replacement therapy; PFC, Pre-frontal cortex*.

It was also shown that intensive and goal-based rehabilitation treatments allow a reduction in dyskinesias (Frazzitta et al., 2012a,c): data from animal models show that in PD striatal plasticity is lost, but chronic L-DOPA treatment is able to restore the long-term potentiation of synaptic transmission (Centonze et al., 2001). The reversal of synaptic strength from the potentiated levels is called depotentiation, and this learning process represents a mechanism for erasing unnecessary motor information. In L-DOPA-induced dyskinesias, synaptic depotentiation is lost possibly representing the cellular basis of dyskinesias (Picconi et al., 2003). Exaggerated movements in response to a stimulation of dopaminergic receptors, such as those occurring during dyskinesias, might consequently convey erroneous information to the motor striate circuits. Therefore, when concomitant, competing correct movements are performed (as during rehabilitation treatment), the manifestation of abnormal dyskinetic movements may be attenuated (Frazzitta et al., 2012a,c).

As a matter of fact it seems that DRT on one side, stimulating the dopamine-denervated striatum, and rehabilitation on the other side, promoting the activity-dependent neuroplasticity, are synergistically able to exploit the physiological mechanisms of cortico-striatal plasticity in PD. Nevertheless, in medicated patients, in the early stages of PD, the differences between nigrostriatal and mesolimbic dopaminergic denervation may induce a “hyperdopaminergic state” in the mesocorticolimbic system that alter the reward-related learning mechanisms (Calabresi et al., 2015). This “state” may interfere with synaptic plasticity properly in the area of basal ganglia involved in habit formation (Bowers et al., 2010; Lüscher and Malenka, 2011; Madsen et al., 2012) and could determine the phenomenon of DDS by creating powerful drug-related pathological memories (Hyman, 2005; Robbins et al., 2008; Simola et al., 2013).

The chronic administration of DRT induces also pronounced hyperdopaminergic stimulation in the nigrostriatal system. This non-physiological stimulation represents a critical factor in the development of dyskinesias: in cynomolgus monkeys, Aubert et al. (2005) demonstrated the central role of the dopamine-D1 receptors (D1Rs), that are co-expressed in the direct pathway neurons with dopamine-D3 receptors, in the pathogenesis of L-DOPA-induced dyskinesias. Particularly, they found that the D1R expression and responsiveness are increased after L-DOPA treatment and that the sensitivity of the D1R signaling cascade is enhanced in L-DOPA-induced dyskinesias (Aubert et al., 2005). Since dopamine-D2 receptor levels expressed by medium spiny neurons of the indirect pathway are neither normalized nor increased after L-DOPA treatment, these authors supported the hypothesis of a predominant role for the direct pathway in L-DOPA-induced dyskinesias (Aubert et al., 2005). In this scenario, it is known that increased responsiveness of the D1R machinery to L-DOPA results in augmented synthesis of cyclic adenosine monophosphate (cAMP), hyper-activation of cAMP-dependent protein kinase (PKA) and cAMP-dependent phosphoprotein of 32 kDa (DARPP-32; Feyder et al., 2011). Abnormal PKA/DARPP-32 signaling increases the phosphorylation of glutamate receptor 1 (Feyder et al., 2011). This effect promotes the excitability of striatal medium spiny neurons and may participate in the loss of corticostriatal long-term depression and depotentiation. Further, sensitized D1R-mediated transmission leads also to activation of extracellular signal-regulated kinases (ERK) and the mammalian target of rapamycin complex 1 (mTORC1), which control transcriptional and translational processes (Santini et al., 2010; Feyder et al., 2011). As in a vicious cycle, all these effects related to this persistent and excessive stimulation of D1Rs exacerbate the dyskinetic motor behavior.

Therefore, while DDS is related to affected striatal reward-related motivational processing, dyskinesias and motor fluctuations represent the inability of striatal neurons to dynamically gate correct cortically driven motor commands (Cenci and Konradi, 2010). This worsens striatum-dependent learning functions (Feigin et al., 2003) with detrimental effects on motor performances. Contrariwise, as previously said, DRT ameliorates cognitive flexibility (Cools, 2006). Thus, dopaminergic neurons regulates cognition in according to an “inverted-U” shaped function, so that too little or too much activity has detrimental effects on executive functions and learning performances depending on the specific task demands (Cools, 2006). All these functional, neuropsychological and biological findings highlight the need for careful DRT titration. Warren Olanow et al. (2013) found a relative L-DOPA threshold effect, with a marked increase in the risks of developing dyskinesias and wearing-off at L-DOPA doses ≥400 mg/day. Based on the results from this study, clinicians should initiate L-DOPA treatment with low doses, proceed by small increments and avoid a total L-DOPA amount >400 mg/day (Warren Olanow et al., 2013).

In addition, the association between DAs dosage and the emergence of pathologic behaviors (Kelley et al., 2012) must be taken into account in the clinical practice. Also in this case, the discontinuation of the DAs or significant adjustment in dosage is the mainstay of treatment intervention (Mamikonyan et al., 2008).

The dopaminergic drugs titration is fundamental with respect to rehabilitation: high levels of DRT induce motor and behavioral side effects and a related negative impact on learning and reward mechanisms, reducing the possibility to achieve good rehabilitative results. Considering that, not disease duration, but DRT dosage accounts for the effectiveness of medication in reducing the reward-based learning (van Wouwe et al., 2012), it is important to promote a synergistic and complementary action between DRT and rehabilitative efforts. PD patients should initiate the rehabilitative treatment as soon as possible and the clinicians should prescribe the “optimal” drug dose. This is fundamental to exploit the DRT efficacy in restoring the abnormal cortico-striatal plasticity (fundamental for skills building) without exhibiting the above-mentioned long-term dopaminergic side effects that are detrimental for rehabilitation.

This is critical for optimal PD management. A more comprehensive study of the interactions between disease, DRT, learning and reward mechanisms is required to deepen the understanding about a complex treatment of a disorder such as PD.

## Author Contributions

DF: provided substantial contributions to discussion of the content, researched data for the article, wrote the text and edited the article before the submission, AC: provided substantial contributions to discussion of the content and researched data for the article, FSU generated table/figure, contributed to the discussion and researched data for the article, GP: researched data for the article and wrote the text, PO: provided substantial contributions to discussion of the content and wrote the text, RM: provided substantial contributions to discussion of the content, MY: provided substantial contributions to discussion of the content and researched data for the article, GF: researched data for the article, wrote the text, provided substantial contributions to discussion of the content and gave the final approval before the submission.

## Conflict of Interest Statement

AC received a consultation fee for a written report for a law firm representing the claimants as part of a class action in Australia against one of the pharmaceutical companies that manufacture a Parkinson’s medication associated with compulsive behavior; Dr. AC reports personal fees from Arnold, Thomas Becker Layers, outside the submitted work; AC is supported by an ARC Discovery Early Career Award (#DE140101097); MY supported by an NHMRC Fellowship Award #APP1021973.
